# Expression of lymphocyte activation markers of preterm neonates is associated with perinatal complications

**DOI:** 10.1186/s12865-016-0159-7

**Published:** 2016-06-21

**Authors:** Florentina Sava, Gergely Toldi, András Treszl, Júlia Hajdú, Ágnes Harmath, Tivadar Tulassay, Barna Vásárhelyi

**Affiliations:** First Department of Obstetrics and Gynecology, Semmelweis University, Baross u. 27, H-1088 Budapest, Hungary; First Department of Pediatrics, Semmelweis University, Budapest, Hungary; MTA-SE Research Group of Pediatrics and Nephrology, Hungarian Academy of Sciences, Budapest, Hungary; Department of Laboratory Medicine, Semmelweis University, Budapest, Hungary

**Keywords:** CD25, CD62L, CD69, Gender, HLA-DR, Preeclampsia, Premature rupture of membranes

## Abstract

**Background:**

Inappropriate activation of T lymphocytes plays an important role in perinatal complications. However, data on T lymphocyte activation markers of preterm infants is scarce. We investigated the association between gender, gestational and postnatal age, preeclampsia (PE), premature rupture of membranes (PROM) as well as prenatal steroid treatment (PS) and the frequency of activated T lymphocyte subsets (HLA-DR+, CD69+, CD25+, CD62L+) and major T lymphocyte subpopulations (CD4, CD8, Th1, Th2, naïve, memory) in peripheral blood during the first postnatal week in preterm infants.

**Results:**

Cord blood and peripheral blood samples were collected from 43 preterm infants on the 1st, 3rd, and 7th days of life. We assessed the frequency of the above T lymphocyte subsets using flow cytometry. The ‘mixed effect model’ was used to analyze the effects of clinical parameters on T lymphocyte markers. The frequency of CD25+ T lymphocytes was higher in PROM. The frequency of CD4+ and CD8+ cells and the CD4+/CD8+ cell ratio was decreased in PE. The frequency of CD62L+ T lymphocytes was higher in male compared with female infants. PS did not affect the frequency of the investigated markers. CD4+ CD25+ cells had a lower frequency at birth than on day 7. Th2 lymphocytes had a lower frequency on postnatal days 1 and 3 when compared to day 7.

**Conclusions:**

Our observations indicate that alterations affecting the expression of T lymphocyte activation markers are associated with the above factors and may play a role in the development of perinatal complications.

## Background

T lymphocyte activation is a finely regulated, complex, cascade of events that result in the expression of cytokine receptors, production and secretion of cytokines, upregulation of cell surface molecules, and activation of direct cell killing. Therefore, it is one of the cornerstones of the implementation of an appropriate immune response for different types of antigens [1].

Preterm birth (birth before the 37^th^ week of gestation) affects an estimated number of 15 million infants each year globally [2]. The rate of preterm birth ranges from 5 to 18 % percent. Premature babies face numerous acute and chronic complications, including respiratory, cardiovascular, gastrointestinal, and perhaps most importantly, neurodevelopmental problems. Risk factors associated with preterm labor include previous preterm births or miscarriages, multiple pregnancy, in vitro fertilization, cervical or placental insufficiency, smoking, poor nutrition, hypertensive disorders, infections, stress and trauma. Numerous studies to date have shown that preterm deliveries associated with preeclampsia (PE), premature rupture of membranes (PROM), intrauterine infection and respiratory distress syndrome (RDS) are linked with higher levels of neonatal adaptive immune response [3]. However, data on T lymphocyte activation markers of preterm infants is scarce [4].

The most important activation molecules expressed on T lymphocytes can be classified as early activation markers, such as CD69 and CD25, and late activation markers, such as CD62L and HLA-DR. Additionally, very late activation markers, such as VLA-1 have also been described, playing a role in lymphocyte adhesion and extravasation [5].

CD69 is regarded as the earliest cell surface activation marker of both umbilical cord and peripheral blood mononuclear cells. Activated neutrophils and eosinophils can also express CD69, therefore it is not restricted to activated lymphocytes. Bone marrow myeloid precursors, platelets and epidermal Langerhans cells also express CD69 constitutively. The engagement of CD69 can activate NK and T cells, resulting in increased cytotoxic activity and pro-inflammatory cytokine production [6]. CD69 seems to be expressed in higher levels on the surface of activated neonatal cells when compared to adults [7]. Upregulation of CD69 on NK cells was identified as a sensitive marker of neonatal infection [8].

CD25, or the alpha subunit of the IL-2 receptor, is involved in the early stage of lymphocyte activation, but it also seems to be critical in maintaining self tolerance and immune homeostasis. Early work on CD4+ CD25 high + cells later termed as regulatory T cells showed that their activation via their T cell receptor (TCR) generates non-specific suppressor cells that suppress the activation of any CD4+ or CD8+ T cell [9]. FoxP3+ regulatory T cells, also characterized by high expression of CD25 are present at the fetal-maternal interface and are important for the maternal acceptance of the allogeneic fetus [10].

CD62L (L-selectin) is considered a late activation marker and a key regulator of T cell trafficking. It acts as a homing receptor for lymphocytes to enter secondary lymphoid tissues via high endothelial venules (HEV). Following activation, CD62L is rapidly downregulated on T cells, which prevents effector T cells from trafficking to lymph nodes through HEV [11]. CD62L is activated during the first postnatal days in preterm infants with RDS, and this activation is associated with the development of bronchopulmonary dysplasia (BPD) [12]. Furthermore, it was also demonstrated that carriers of the L-selectin 213Ser allele are at increased risk for premature birth and BPD [13].

HLA-DR molecules are important in antigen processing and presentation, mediating antigen-specific T cell activation [14]. It is known that low levels of HLA-DR expression on monocytes contributes to impaired neonatal host defense, especially in preterm infants [15]. Decreased expression of HLA-DR molecules in preterm newborns is linked with development of several complications, such as high incidence of bacterial infections and pulmonary morbidity, especially in the presence of RDS [16, 17].

The aim of this study was to assess the association of gender, gestational and postnatal age, preeclampsia (PE), premature rupture of membranes (PROM) and prenatal steroid treatment (PS) with the frequency of activated T lymphocyte subsets (CD69+, CD25+, CD62L+, HLA-DR) and major T lymphocyte subpopulations (CD4, CD8, Th1, Th2, naïve, memory) in peripheral blood during the first postnatal week in preterm neonates. Since data on the physiological frequency of these cell subsets is challenging to obtain, we aimed to gather preliminary data to describe the dynamic postnatal alteration of these parameters in preterm neonates affected by different perinatal factors.

## Methods

### Patients

We enrolled 43 preterm infants (22 female and 21 male) in this study. Gestational age was 30 (25–33) weeks, while birthweight was 1300 (490–1980) g at birth. The suspected ground for preterm birth was PE in 8 cases, PROM in 13 cases, and could not be settled in 22 cases. PS treatment was applied in 25 cases. All infants had a highly suspected or proven intrauterine infection based on standard criteria [18, 19]. PROM cases were coupled with elevated IL-6 levels (256.2 (64.8–2358.9) pg/ml) measured in cord blood, while IL-6 levels in cord blood of infants who had no PROM were normal (11.6 (2.9–45.1) pg/ml). Patient characteristics are summarized in Table 1.

**Table 1:** Clinical characteristics of preterm neonates enrolled in the study

Gestational age (weeks)	30 (25–33)
No. of infants born before 29th week	13 (30 %)
No. of infants born on 29-30th week	15 (35 %)
No. of infants born after 30th week	15 (35 %)
Birth weight (grams)	1300 (490–1980)
Apgar score at 1 min	8 (5–9)
Apgar score at 5 min	9 (7–10)
No. of male infants	21 (49 %)
No. of neonates born by Cesarean section	26 (60 %)
No. of neonates with maternal steroid prophylaxis	25 (58 %)
No. of neonates with suspected intrauterine infection	43 (100 %)
No. of preeclampsia	8 (19 %)
No. of premature rupture of membranes	13 (30 %)

Data are presented as median (range)

Written informed consent was obtained from parents of subjects, and our study was reviewed and approved by an independent ethical committee of the institution (Semmelweis University, Budapest). The study was adhered to the tenets of the most recent revision of the Declaration of Helsinki.

### Sample collection

Cord blood mononuclear cells (CBMCs) were separated by a standard density gradient centrifugation (Ficoll Paque, Amersham Biosciences AB, Uppsala, Sweden, 25 min, 400 g, 22 °C) from freshly drawn blood collected in lithium heparin-treated tubes (BD Vacutainer, BD Biosciences, San Jose, CA, USA). Peripheral blood samples were taken on the 1st, 3rd, and 7th postnatal days of life.

### Flow cytometry

**Fig. 1:**
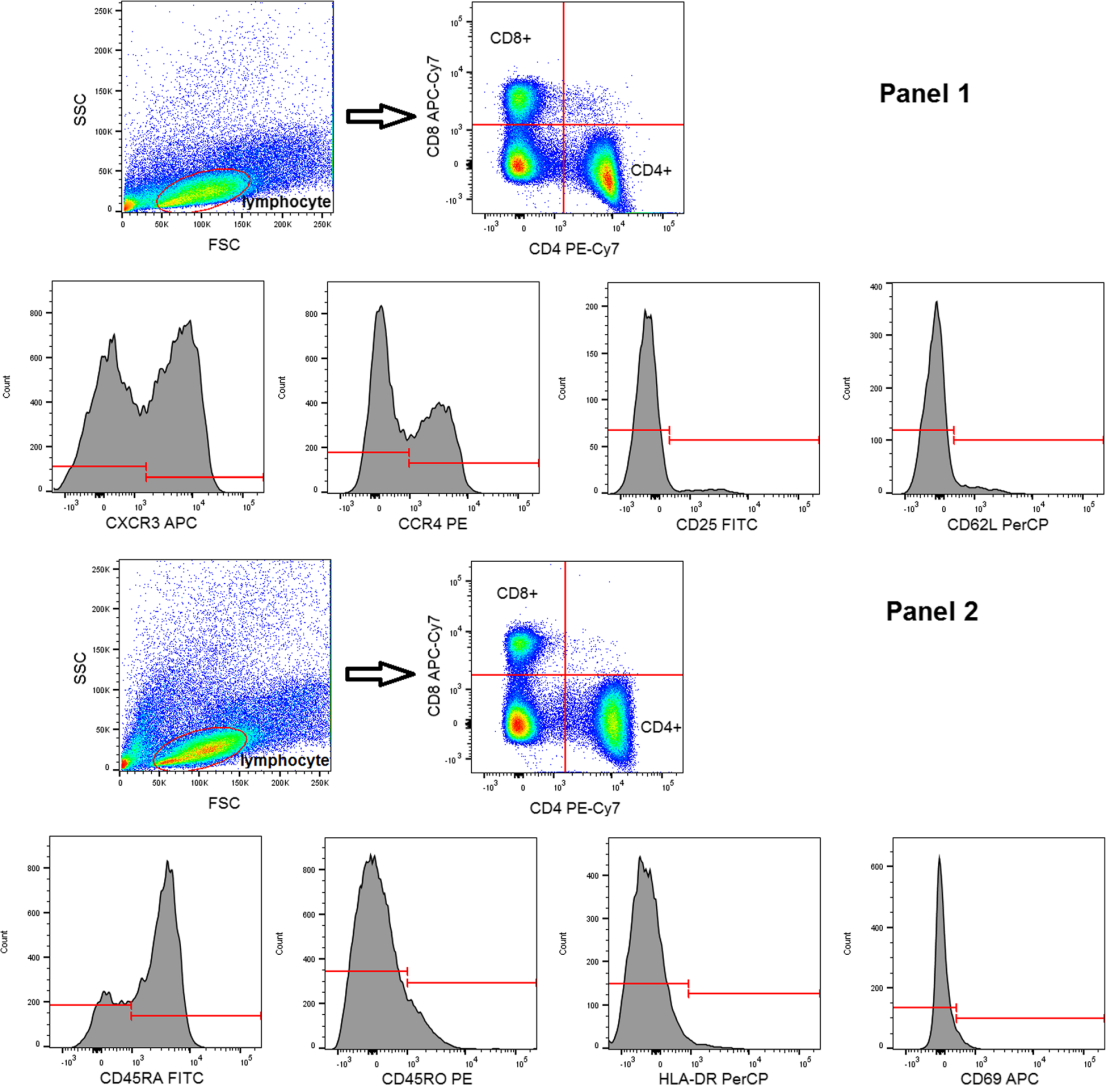
Gating strategy of flow cytometry measurements. Example of a representative sample. FSC – forward scatter, SSC – side scatter

CBMCs and peripheral whole blood were stained for 30 min at room temperature in the dark with the following monoclonal antibodies: PE Cy7-conjugated CD4, APC-Cy7-conjugated CD8, FITC-conjugated CD25, PerCP-conjugated CD62L, APC-conjugated CXCR3, PE-conjugated CCR4, APC-conjugated CD69, PerCP-conjugated HLA-DR, FITC-conjugated CD45RA, PE-conjugated CD45RO in separate tubes, respectively (all from BD Biosciences). After lysing red blood cells and washing, CBMCs and PBMCs were analyzed on a BD FACSAria flow cytometer (BD Biosciences) equipped with 488 nm and 633 nm excitation lasers. Data were processed using the FACSDiVa software. Figure 1 demonstrates the gating strategy applied.

Th1 cells were defined as CD4+ CXCR3+, while Th2 cells were defined as CD4+ CCR4+. Naïve T cells were defined as CD4+ CD45RA+, while memory T cells were defined as CD4+ CD45RO+.

### Statistics

Data are expressed as median (range). The independent effects of gestational and postnatal age, PE, PROM, PS and gender were analyzed using the ‘mixed effect model’ method. This is a statistical model containing both fixed effects and random effects. It is particularly used in settings where repeated measurements are made on the same statistical units (ie. longitudinal studies), or where measurements are made on clusters of related statistical units [20]. Statistics were calculated at 5 % significance level (*p* = 0.05) using the SAS software (Cary, NC, USA). A nominal *p* value < 0.05 was considered statistically significant. No adjustment for multiplicity was performed, thus some of p values may be spurious.

## Results

**Fig. 2:**
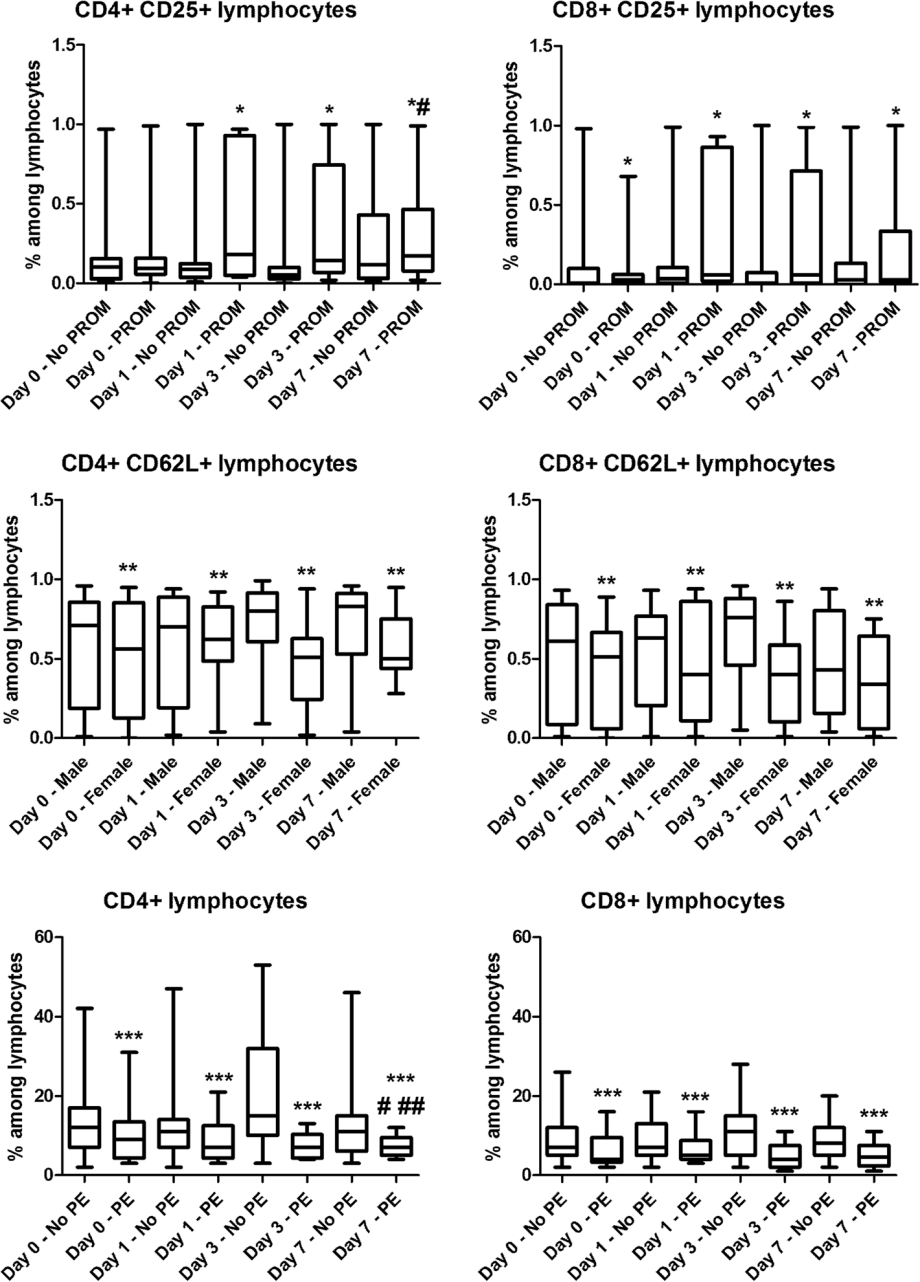
Box-plots representing frequency values of the investigated cell subsets in different subgroups of preterm infants at birth (Day 0) and on days 1, 3 and 7 of life. Horizontal line: median, box: interquartile range, whisker: range. PE – preeclampsia, PROM – premature rupture of membranes. *p < 0.05 vs. No PROM, **p < 0.05 vs. Male infants, ***p < 0.05 vs. No PE, #*p* < 0.05 vs. Day 0, ##*p* < 0.05 vs. Day 0

Results are summarized in Table 2 and Fig. 2.

**Table 2:** Significant results of mixed effect model analysis for the investigated factors. “% change” is expressed vs. Day 7 for postnatal age, vs. PE (present) for preeclampsia, vs. PROM (present) for premature rupture of membranes, vs. Boys for gender, vs. < 29 weeks for gestational age

T cell subset	Effect	*p*	Estimate	% change
CD4+	Day 0	0.0487	0.04143	4
	Day 3	0.0018	0.06641	6
	No PE	0.023	0.09089	9
CD8+	No PE	0.0371	0.02683	2
CD4+ CD25+	Day 0	0.0331	−0.1305	−87
	No PROM	0.0219	−0.1826	−83
CD8+ CD25+	No PROM	0.0285	−0.1592	−86
CD4+ CD62L+	Boys	0.0572	0.1071	10
CD8+ CD62L+	Boys	0.0309	0.1404	15
CD4+ CXCR3+	29-30 weeks	0.0291	−0.1256	−88
CD4+ CCR4+	Day 1	0.0341	−0.1342	−87
	Day 3	0.024	−0.1431	−86

The frequency of CD4+ CD25+ and CD8+ CD25+ activated T lymphocytes was higher in cases with PROM at all time points. We observed a decrease in the frequency of CD4+ and CD8+ T lymphocytes as well as the CD4+/CD8+ T cell ratio in PE compared to infants not affected by PE at all time points. The frequency of CD4+ CD62L+ and CD8+ CD62L+ T lymphocytes was higher in male infants when compared to female infants at all time points.

None of the investigated factors had an effect on the expression of the HLA-DR and CD69 activation markers, or the frequency of Th1 (CD4+ CXCR3+), Th2 (CD4+ CCR4+), naïve (CD45RA+) and memory (CD45RO+) T cell subsets.

The frequency of Th1 (CD4+ CXCR3+) lymphocytes was higher in infants born before the 29th gestational week compared to those born on the 29-30th gestational week on postnatal days 1 and 3.

When we looked at the effect of postnatal age (day 1, 3 and 7 of life) on the frequency of the investigated markers and subsets, we detected several changes. CD4+ T cells have a higher frequency on postnatal days 0 and 3 when compared to day 7. CD4+ CD25+ cells had a lower frequency on postnatal day 0 than on day 7. Of note, Th2 (CD4+ CCR4+) lymphocytes also had a lower frequency on postnatal days 1 and 3 when compared to day 7.

## Discussion

Both prenatal and postnatal inflammation are important factors in the pathogenesis of many adverse outcomes in preterm infants. An important feature of the inflammatory response is T lymphocyte activation and the expression of early and late activation markers on T cells. Luciano et al. demonstrated that preterm deliveries are associated with higher levels of T cell activation markers, such as CD25, HLA-DR, and CD69 compared to term deliveries. In their study, clinical chorioamnionitis was also associated with an increase in T cell activation markers. Their findings support that fetal adaptive immune activation in utero is closely associated with preterm labor [4].

Our study shows that the frequency of CD4+ CD25+ and CD8+ CD25+ activated T lymphocytes is higher in cases with PROM. Similarly to other obstetrical pathologies, the etiology of PROM is multifactorial. However, there is evidence suggesting that subclinical intrauterine infection is a major factor in the pathogenesis of PROM [21]. The pathogens ascending into the decidua and entering the fetal membranes generate a cascade of maternal and fetal inflammatory responses that finally result in membrane weakening and rupture [22]. In our patient population, PROM was also associated with an elevation of cord blood IL-6 levels, indicating ongoing inflammation probably due to intrauterine infection in these infants. The increased expression of CD25 on neonatal T lymphocytes might be another representation of this inflammatory response.

PE is a major cause of fetal and maternal morbidity and mortality and is recognized as a multisystem disorder of human pregnancy. Although maternal immunological alterations, such as an increase in the Th17/Treg ratio [23], are relatively well described in PE, very limited information is available on how this disorder affects the fetal/neonatal immune system. In our current study, we observed a decrease in the frequency of CD4+ and CD8+ T lymphocytes as well as the CD4+/CD8+ T cell ratio in PE compared to infants not affected by PE. These findings are in line with previous results [3]. However, no clear cause of this phenomenon has been identified. Theoretically, intrauterine malnutrition that often affects fetuses in PE pregnancies, may be a factor delaying or inhibiting maturation if immune cell types.

The frequency of CD62L+ or L-selectin expressing T lymphocytes was higher in male infants when compared to female neonates in our study. Previous investigations demonstrated that the altered expression and polymorphisms of selectins are related to prematurity and BPD [13]. Male vulnerability has been previously noted in infants. Males had more postnatal complications compared to females, including lower Apgar scores, higher supplemental oxygen need, higher rates of RDS, a poor neurological outcome at follow-up, and a higher overall perinatal mortality [24]. It has also been suggested that lung immaturity in premature boys contributes to their poorer outcome [25]. Thus our results might indicate that the elevated morbidity of male infants is closely linked to a higher frequency of CD62L+ lymphocytes.

This is also in line with findings of Turunen et al. who demonstrated that RDS is associated with a lower T cell count and a higher frequency of CD62L expressing cells. The authors concluded that increased frequency of activated T cells predicts the development of BPD, and systemic T cell activation could mediate inflammation contributing to its pathogenesis [12].

Prenatal steroids undisputedly decrease neonatal morbidity and mortality by improving fetal lung maturation. The thymus is essential for the development and selection of T cells, and thymocytes are very sensitive to steroids [26]. In the current study, PS treatment did not affect the frequency of lymphocyte activation markers during the first postnatal week of life. Thus, based on our results, PS does not exert an immunomodulatory effect on the frequency of activated lymphocytes investigated in this study.

Maturation of the adaptive immune responses occurs mostly after birth. Activated CD25+ CD4+ T cells had a lower frequency at birth when compared to day 7 of life, probably due to the lack of antigenic stimulation from the environment in utero. Further studies are needed to elucidate the effects of the transition occurring after birth.

Th2 lymphocytes appeared to have a lower frequency on postnatal days 1 and 3 when compared to day 7. Cytokine responses are skewed towards the Th2 direction in the fetus. This bias is thought to contribute to the prevention of fetal rejection by the maternal immune system [27]. Preterm and term neonates are thought to be vulnerable to infection due to this bias to a Th2 phenotype [28]. However, parturition, independently of the presence of infection, is associated with a marked Th1 response. This might be represented by lower Th2 cell numbers directly after birth in preterm infants on days 1 and 3 of life.

## Conclusions

In conclusion, the frequency of CD25+ activated T lymphocytes was higher in cases with PROM. The frequency of CD4+ and CD8+ T lymphocytes as well as the CD4+/CD8+ T cell ratio was lower in infants affected by PE. The frequency of CD62L+ T lymphocytes was higher in male infants.

Our observations indicate that alterations affecting the expression of early and late T lymphocyte activation markers in preterm infants are associated with the presence of PROM, PE, and gender, and may play a role in the development of perinatal complications.

## Abbreviations

BPD, bronchopulmonary dysplasia; CBMC, cord blood mononuclear cell; HEV, high endothelial venules; NK, natural killer cell; PE, preeclampsia; PROM, premature rupture of membranes; PS, prenatal steroid; RDS, respiratory distress syndrome; TCR, T cell receptor
